# Giant cell tumor of the distal ulna: a case report

**DOI:** 10.1186/1752-1947-6-143

**Published:** 2012-06-01

**Authors:** Daniele Vanni, Andrea Pantalone, Elda Andreoli, Patrizio Caldora, Vincenzo Salini

**Affiliations:** 1 Orthopedic and Traumatologic Division, “G. d’Annunzio” University, Via dei Vestini 35, 66013, Chieti, Italy; 2 Orthopedic and Traumatologic Division, “S. Margherita” Hospital, Via N.A. Fratta 145, 52040, Cortona, Arezzo, Italy

**Keywords:** Adjuvant therapy, Curettage, GCT, Phenol, Ulna

## Abstract

**Introduction:**

Several cases of long bone giant cell tumor have been reported in the literature. We report the case of a patient with a giant cell tumor in the distal ulna. This is very unusual, with a reported incidence of 0.45 to 6%.

**Case presentation:**

A 17-year-old Colombian man presented with a painful swelling of the left wrist. After performing an instrumental examination, a diagnosis of distal ulna giant cell tumor was made. The tumor was treated with an intralesional curettage, phenol application and bone grafting.

**Conclusions:**

This tumor may have a good prognosis if it is diagnosed early and radically treated. It is important to be aware of atypical cancer localizations in order to perform a proper diagnosis.

## Introduction

Bone giant cell tumor (GCT) is a rare, generally benign and locally aggressive tumor. It represents approximately 3% to 5% of all primary bone cancers. It generally occurs in adults between the ages of 20 and 40 years. GCT of bone is very rarely seen in children or in adults older than 65 years of age. GCT tumors occur in approximately one person per million per year. Usually, the tumor site is at the long bone meta-epiphysis, especially the distal radius and femur, proximal humerus and tibia. The ulna distal extremity is an unusual site (0.45% to 3.2%) for a primary bone GCT. We report the case of a distal ulna GCT diagnosed in a 17-year-old man. It was treated with intralesional curettage, adjuvant therapy with 5% phenol and a synthetic bone graft reconstruction.

## Case presentation

**Figure 1:**
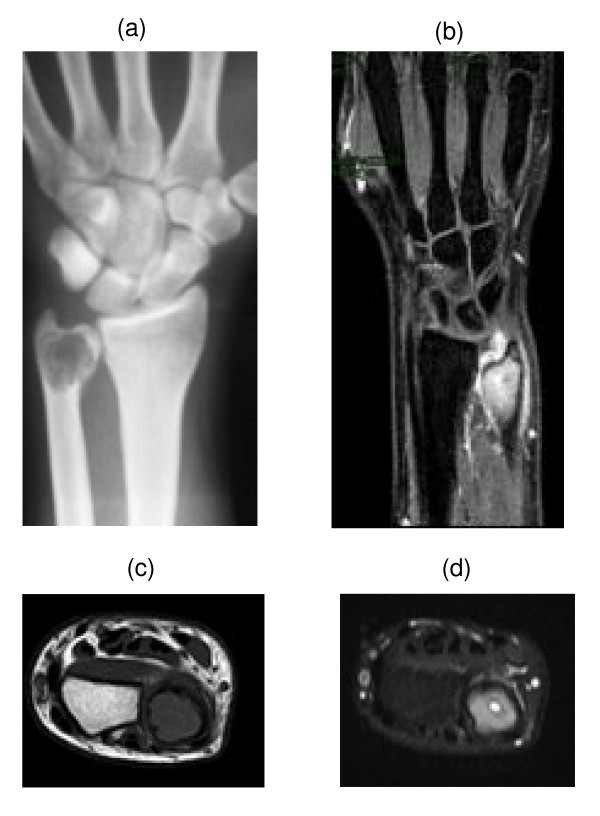
X-rays and magnetic resonance imaging with and without contrast.

A radiographic examination of the left wrist was performed in a 17-year-old Colombian man as a result of a direct incidental trauma. No fracture was seen, but an osteolytic area was found in the ulnar meta-epiphysis. The initial diagnosis was ‘juvenile bone cyst’. The patient presented with a painful swelling at the wrist dorsal ulnar side, about 2.5 cm, in the absence of any epidermal dyschromias. The skin was elastic and smooth. Wrist examination showed a range of motion (ROM) of 45 ° of extension, 70 ° of flexion, 15 ° of radial deviation and 10 ° of ulnar deviation; pronation, supination and circumduction were painful. Contralateral wrist ROM was normal. The diagnosis of ‘juvenile bone cyst’ did not seem right and a second radiographic examination was performed. At this time, a multilocular osteolytic area inducing an expansion of the distal ulna was seen. A cortical bone interruption was also visible. Although the patient had been the victim of a trauma, this was a poor prognostic sign. Therefore an Magnetic resonance imaging examination was performed. It showed a hypointense signal in the T1 sequences and a hyperintense signal in the short TI inversion recovery (STIR) sequences, characterized by enhancement after contrast administration, due to the presence of newly formed tissue (Figure 1).

After an incisional biopsy, the diagnosis of GCT was finally made, although it was in an atypical site.

**Figure 2:**
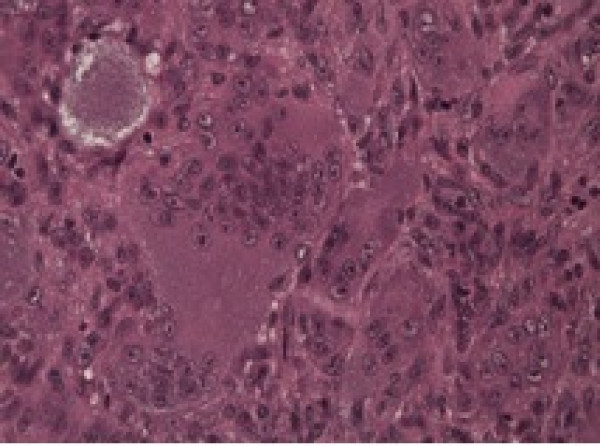
Mononuclear stromal cells associated with osteoclast-like multinucleated giant cells evenly distributed.

The lesion was classified as a grade II with fracture (Figure 1), according to the Campanacci Classification and as a stage II according to the Enneking Classification for benign bone tumors (Figure 2).

Subsequently, the patient was surgically treated with intralesional curettage, adjuvant therapy with 5% phenol and a synthetic bone graft reconstruction.

A cortical bone rawplug was removed through a skin incision at the dorsomedial side of the deformity. Accessing the cystic cavity revealed a gelatinous ‘chocolate brown’ material, with many areas of darker color.

**Figure 3:**
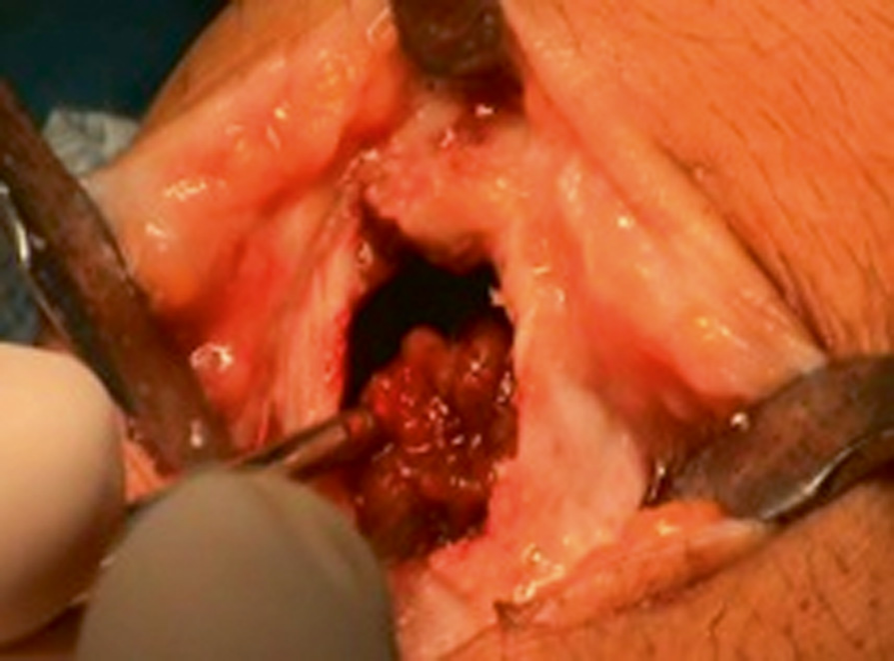
Emptying of giant cell tumor.

**Figure 4:**
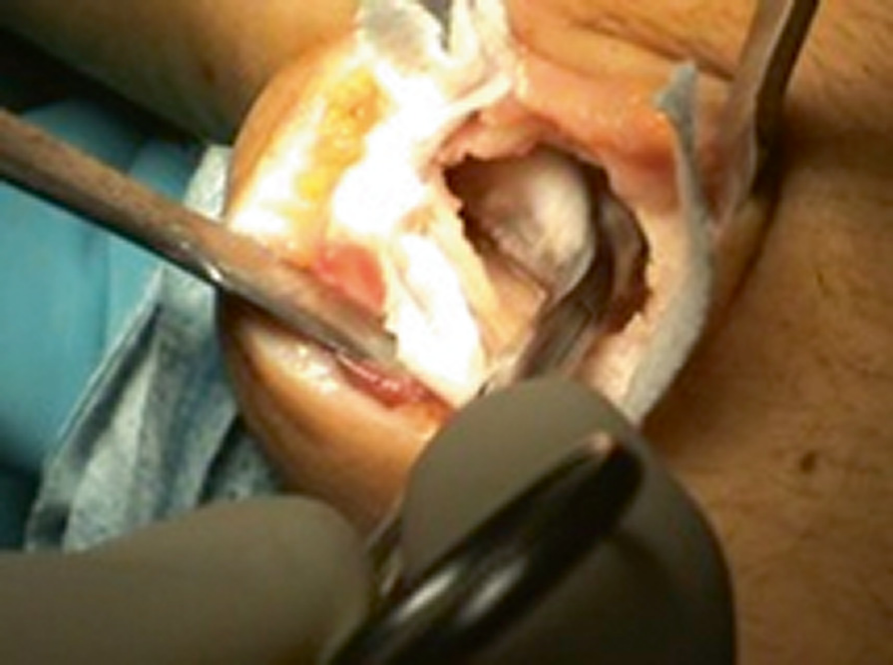
Curettage, adjuvant administration of 5% phenol and subsequent neutralization with hydrogen peroxide.

The cavity was carefully emptied and curetted (Figure 3), and then 5% phenol was applied in three cycles (Figure 4), with subsequent neutralization by hydrogen peroxide.

**Figure 5:**
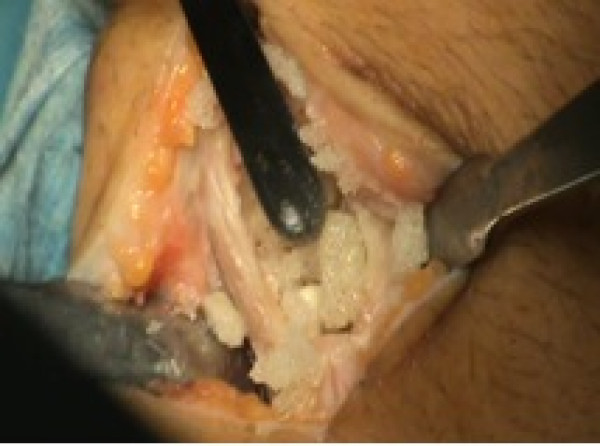
Reconstruction with synthetic bone.

**Figure 6:**
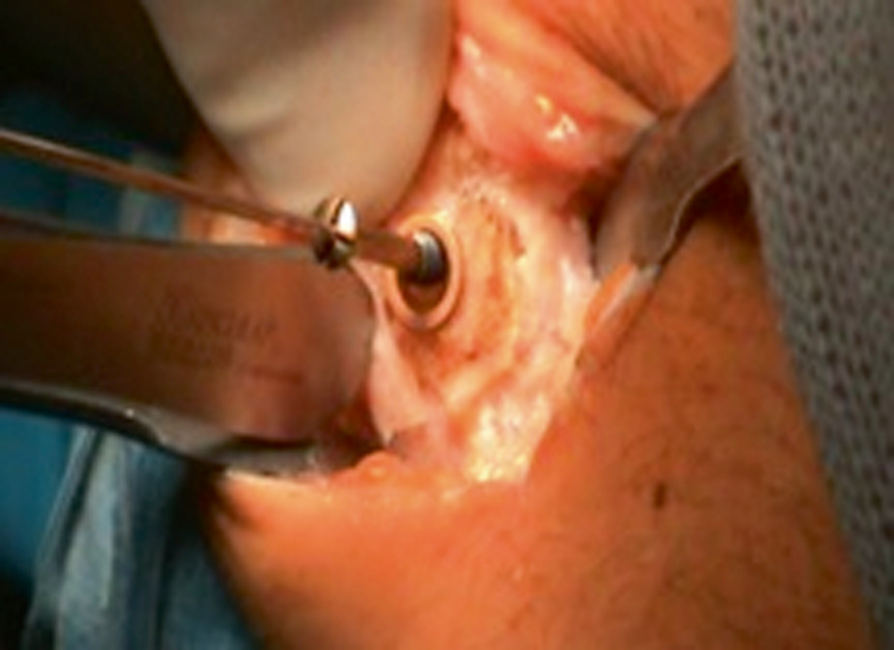
Osteosynthesis and stabilization with a cannulated screw.

**Figure 7:**
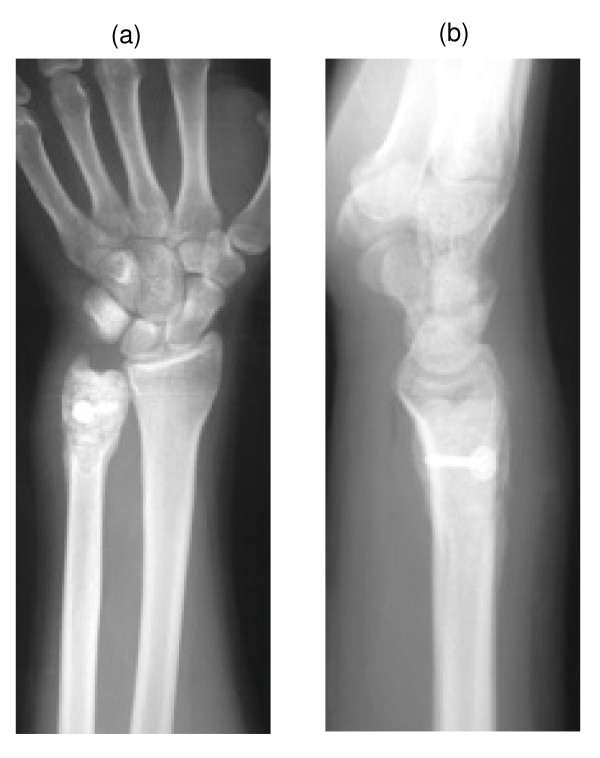
a-b: Post-operative radiology.

Then, using synthetic cancellous bone substitutes, a bone defect fill was performed (Figure 5) and the bone rawplug was synthesized using a cannulated screw (Figure 6, Figure 7).

A clinical and radiographic follow-up was performed one, three, six, 12, 24 and 48 months post-operatively to evaluate the bone consolidation. Chest radiographs were performed every six months. Within the first post-operative month, the patient completely recovered the wrist ROM. After 3 years, there have been no signs of recurrence.

## Discussion

The bone GCT was first described in 1818 by Cooper and Travers. Its local aggression has been highlighted by Nelaton and its malignant potential by Virchow.

It is a rare tumor, essentially benign, but it may behave unexpectedly, regardless of the results of radiological or histological examinations.

It is usually located in the long bone meta-epiphysis and it frequently involves the subchondral bone without involvement of the articular surface; however, larger tumors may extend into the metaphysis and, more rarely, into the diaphysis. Proximal tibia, humerus, distal femur and radius are typical sites.

GCT represents about 3% to 5% of all bone tumors and 21% of benign bone tumors [1,2].

In 70% of cases, it involves women in the third to fourth decade of life.

The distal epiphysis of the ulna is an unusual place for a primary bone GCT; in fact, this occurs in only 0.45% to 3.2% of all primary bone GCT’s [3].

In the past, these tumors were treated with amputation or large resections and ulterior reconstructions. Currently, surgical treatments are:

· Intralesional curettage

· Curettage and bone grafting

· Cryotherapy of the cavity after curettage

· Application of phenol after curettage

· Radiation

· Insertion of methyl methacrylate cement in the cavity after curettage

· Resection followed by allograft

· En-bloc resection with or without reconstruction or stabilization of the ulna and prosthetic reconstruction

· Embolization of the feeding vessels

The variables related to the tumor, such as size, location, biological activity, cortical bone destruction or pathologic fracture evidence, determine the treatment [4].

Although an en-bloc resection radically assaults the tumor, significantly reducing the risk of recurrence, functional outcome is very bad. A simple curettage provides an excellent functional outcome, but with a higher recurrence rate of approximately 40% [1-5] if compared with the patients who received adjuvant therapy (45% versus 18%).

Therefore, various adjuvant therapies have been associated with the curettage: phenol, cryotherapy [6-8], cement or polymethyl methacrylate (PMMA) used intraoperatively.

The recurrence rate ranges from 5% to 8% when cement is used, and approximately 2.3% after cryosurgery [6,7].

However, it needs to be mentioned that a multicenter study of the Canadian Sarcoma Group [9] reported an overall recurrence rate of 17% and claimed that the filling material or the type of adjuvant would not have an absolute impact on recurrence.

Furthermore, some studies show that the use of an adjuvant would not be necessary in some cases, such as intraosseous GCT [10].

According to Schajowicz [11], curettage alone is an inadequate oncological procedure, but when it is combined with an adjuvant therapy, it globally provides a better result with respect to one-block excision, especially in terms of functionality.

Therefore, the correct treatment must achieve a balance between oncological radicality and the restoration of skeletal segment functionality [12-14].

Curettage associated with bone grafting has been shown to be effective in many cases [15]. In this study it is used with phenol as an adjuvant, because it is capable of causing protein and DNA coagulation, inducing cell necrosis.

In the present case intralesional curettage was possible because the tumor was a grade II and the reconstruction was carried out with synthetic cancellous bone, due to the young age of the patient.

## Conclusions

Neoformation bone diagnosis is difficult and requires a great deal of experience, especially in young patients. Osteolytic lesions incidentally found at a long bone epiphysis, can be misinterpreted. This tumor may have a good prognosis if treated early and radically. It is important to know atypical cancer locations in order to perform a proper diagnosis.

## Consent

Written informed consent was obtained from the patient’s next-of-kin for publication of this case report and any accompanying images.

## Competing interests

The authors declare that they have no competing interests.

## Authors’ contributions

DV was a major contributor in writing the manuscript and was involved in the bibliographic research. AP was involved in the bibliographic research and was also a major contributor in writing the manuscript. EA performed the surgery and was involved in the bibliographic research. PC performed the surgery and contributed in writing the manuscript. VS also performed the surgery and contributed in writing the manuscript. All authors read and approved the final manuscript.

## References

[B1] Beebe-Dimmer JL Cetin K Fryzek JP Schuetze SM Schwartz K The epidemiology of malignant giant cell tumors of bone: an analysis of data from the Surveillance, Epidemiology and End Results Program (1975–2004) Rare Tumors 2009 1 e52 21139931 10.4081/rt.2009.e52 PMC2994468

[B2] Campanacci M Baldini N Boriani S Sudanese A Giant-cell tumor of bone J Bone Joint Surg Am 1987 69 106 114 3805057

[B3] Goldenberg RR Campbell CJ Bonfiglio M Giant-cell tumor of bone. An analysis of two hundred and eighteen cases J Bone Joint Surg Am 1970 52 619 664 5479455

[B4] Sung HW Kuo DP Shu WP Chai YB Liu CC Li SM Giant-cell tumor of bone: analysis of two hundred and eight cases in Chinese patients J Bone Joint Surg Am 1982 64 755 761 7045129

[B5] Masui F Ushigome S Fujii K Giant cell tumor of bone: a clinicopathologic study of prognostic factors Pathol Int 1998 48 723 729 10.1111/j.1440-1827.1998.tb03973.x 9778111

[B6] Malawer MM Bickels J Meller I Buch RG Henshaw RM Kollender Y Cryosurgery in the treatment of giant cell tumor. A long-term followup study Clin Orthop Relat Res 1999 359 176 188 10078141 10.1097/00003086-199902000-00019

[B7] Malawer MM Marks MR McChesney D Piasio M Gunther SF Schmookler BM The effect of cryosurgery and polymethyl methacrylate in dogs with experimental bone defects comparable to tumor defects Clin Orthop Relat Res 1988 226 299 310 3335103

[B8] Marcove RC Weis LD Vaghaiwalla MR Pearson R Huvos AG Cryosurgery in the treatment of giant cell tumors of bone. A report of 52 consecutive cases Cancer 1978 41 957 969 10.1002/1097-0142(197803)41:3<957::AID-CNCR2820410325>3.0.CO;2-Y 638982

[B9] Turcotte RE Wunder JS Isler MH Bell RS Schachar N Masri BA Moreau G Davis AM Canadian Sarcoma Group Giant cell tumor of long bone: a Canadian Sarcoma Group study Clin Orthop Relat Res 2002 397 248 258 11953616 10.1097/00003086-200204000-00029

[B10] Prosser GH Baloch KG Tillman RM Carter SR Grimer RJ Does curettage without adjuvant therapy provide low recurrence rates in giant-cell tumors of bone? Clin Orthop Relat Res 2005 435 211 218 15930941 10.1097/01.blo.0000160024.06739.ff

[B11] Schajowicz F Tumors and Tumor-like Lesions of Bone: Pathology, Radiology and Treatment 1994 Springer-Verlag,

[B12] Gracia I Proubasta IR Trullols L Peiró A Moya E Cortés S Buezo O Majó J Distal radioulnar joint prosthesis for the treatment of giant cell tumor of the distal ulna: a case report and literature review Strategies Trauma Limb Reconstr 2011 6 103 106 10.1007/s11751-011-0113-4 21773775 PMC3150648

[B13] Singh M Sharma S Peshin C Wani IH Tikoo A Gupta SK Singh D Wide resection and stabilization of ulnar stump by extensor carpi ulnaris for giant cell tumor of distal ulna: two case reports Cases J 2009 2 8617 10.4076/1757-1626-2-8617 19830093 PMC2740324

[B14] Burke CS Gupta A Buecker P Distal ulna giant cell tumor resection with reconstruction using distal ulna prosthesis and brachioradialis wrap soft tissue stabilization Hand (N Y) 2009 4 410 414 10.1007/s11552-009-9192-9 PMC2787221 19370378

[B15] Ward WG Li G Customized treatment algorithm for giant cell tumor of bone: report of a series Clin Orthop Relat Res 2002 397 259 270 11953617 10.1097/00003086-200204000-00030

